# Origin and development of oligoadenylate synthetase immune system

**DOI:** 10.1186/s12862-018-1315-x

**Published:** 2018-12-27

**Authors:** Jiaxiang Hu, Xiaoxue Wang, Yanling Xing, Enguang Rong, Mengfei Ning, Jacqueline Smith, Yinhua Huang

**Affiliations:** 1 State Key Laboratory for Agrobiotechnology, China Agricultural University, No.2 Yuan Ming Yuan West Road, Hai Dian District, Beijing, 100193 China; 2 The Roslin Institute and Royal (Dick) School of Veterinary Studies, University of Edinburgh, Edinburgh, UK

**Keywords:** OAS, OASL, dsRNA, Neo-functionalization, Sub-functionalization

## Abstract

**Background:**

Oligoadenylate synthetases (OASs) are widely distributed in Metazoa including sponges, fish, reptiles, birds and mammals and show large variation, with one to twelve members in any given species. Upon double-stranded RNA (dsRNA) binding, avian and mammalian OASs generate the second messenger 2'-5'-linked oligoadenylate (2-5A), which activates ribonuclease L (RNaseL) and blocks viral replication. However, how Metazoa shape their OAS repertoires to keep evolutionary balance to virus infection is largely unknown. We performed comprehensive phylogenetic and functional analyses of OAS genes from evolutionarily lower to higher Metazoa to demonstrate how the OAS repertoires have developed anti-viral activity and diversified their functions.

**Results:**

Ancient Metazoa harbor OAS genes, but lack both upstream and downstream genes of the OAS-related pathways, indicating that ancient OASs are not interferon-induced genes involved in the innate immune system. Compared to OASs of ancient Metazoa (i.e. sponge), the corresponding ones of higher Metazoa present an increasing number of basic residues on the OAS/dsRNA interaction interface. Such an increase of basic residues might improve their binding affinity to dsRNA. Moreover, mutations of functional residues in the active pocket might lead to the fact that higher Metazoan OASs lose the ability to produce 3'-5'-linked oligoadenylate (3-5A) and turn into specific 2-5A synthetases. In addition, we found that multiple rounds of gene duplication and domain coupling events occurred in the OAS family and mutations at functionally critical sites were observed in most new OAS members.

**Conclusions:**

We propose a model for the expansion of OAS members and provide comprehensive evidence of subsequent neo-functionalization and sub-functionalization. Our observations lay the foundation for interrogating the evolutionary transition of ancient OAS genes to host defense genes and provide important information for exploring the unknown function of the OAS gene family.

**Electronic supplementary material:**

The online version of this article (10.1186/s12862-018-1315-x) contains supplementary material, which is available to authorized users.

## Background

RNA viruses pose big challenges to human health due to their rapid replication kinetics and high mutation rates. Host cells interact with RNA viruses through recognizing their pathogen-associated molecular patterns (PAMP), such as dsRNA, and then activate the innate immune response. Oligoadenylate synthetases (OASs) are dsRNA sensors distributed widely in Metazoa. The OAS repertoires show large variation, where one, four and twelve OAS members have been identified in birds, primates and rodents respectively [1–3]. The human genome harbors four OAS family members, namely OAS1, OAS2, OAS3 and OASL1. OAS1/OASL, OAS2, and OAS3 are composed of one, two and three OAS units respectively. Moreover, OASL contains two additional tandem ubiquitin-like domains (UBL) at its C terminus [4]. Expression of OAS genes is up-regulated during infections of a wide spectrum of RNA viruses, such as human immunodeficiency virus (HIV) [5] and influenza A virus [6]. Upon binding dsRNA, the OAS protein undergoes an elaborate conformational rearrangement [7] and the active OAS/dsRNA complex polymerizes ATP into 2′-5′-linked oligoadenylate (2-5A) ranging from dimers up to 30-mers [8]. Trimers or higher oligomers can serve as unique second messengers to activate ribonuclease L (RNaseL) and finally induce RNA degradation [9].

OASs diverge from nucleotidyl transferases (such as poly adenosine polymerases (PAP) and CCA-adding enzymes), which only catalyze the formation of 3'-5'-phosphodiester at the beginning of Metazoa evolution [10]. Marine sponge OAS proteins have been reported as the most ancient enzymatically active OAS proteins in Metazoa. They may represent a link between earlier 3'-specific polymerases (3'-5'-ligase) and 2'-specific OASs (2'-5'-ligase) in higher vertebrates since sponge OASs synthesize both 3-5A and 2-5A [11]. Moreover, sponge OASs act in a dsRNA-independent manner, which is different from higher vertebrate OASs that act in a dsRNA-dependent manner [12]. However, how OASs in higher vertebrates lost their 3′-product synthesis ability and evolved to be 2′-specific and dsRNA sensitive enzymes is largely unknown.

Here, we collected a total of 152 OAS and OASL protein sequences from 89 species, and searched for genes involved in the OAS/RNaseL, OASL/RIG-I (Retinoic acid-inducible gene I) and OASL/IRF7 (Interferon regulatory factor 7) pathways in ancient Metazoa. The data suggested that ancient OASs were not interferon-induced immune genes. We then performed sequence and structure alignment to infer the evolutionary force driving OASs to be dsRNA sensors. Moreover, we constructed phylogenetic trees using these full-length proteins or OAS domains. These analyses present a detailed model showing how the OAS repertoires have been shaped. This study extends our knowledge about the evolutionary pattern of the OAS family and gives insight into their function.

## Methods

### Cell culture, transfections, and quantitative RT-PCR

HeLa (human cervical carcinoma cells) and DF1 (Chicken embryonic fibroblasts cells) were purchased from American Type Culture Collection (ATCC). All cells were grown in Dulbecco's modified Eagle's medium (DMEM) containing 10% fetal bovine serum (FBS, Gibco, Carlsbad, USA) in an atmosphere of 5% CO2 at 37 °C. Transfection of 2-5A was done with Lipofectamine 2000 (Invitrogen, Rockville, USA) according to the manufacturer’s protocol. After 6 h post transfection, the total RNA was isolated from cells using TRIzol reagent (Invitrogen, Rockville, USA). cDNA was synthesized using cDNA Synthesis Kit (Thermo Scientific, Waltham, USA) and used to examine gene expression using primers Table S1 through normalizing the corresponding expression of the GAPDH reference gene (Additional file 1: Table S1).

### Phylogenetic analysis

All sequences were retrieved from the NCBI (https://www.ncbi.nlm.nih.gov/) database. Protein or CDS sequences of human OAS-related genes (OAS1, OAS2, OAS3, OASL, IFN, IFNR, STAT1/2, JAK, RNaseL, RIG-I, IRF7 and TOPI) were used to query against the nr database using default BLAST parameters. All protein sequences were subjected to Interproscan (http://www.ebi.ac.uk/interpro/) analysis with default options to validate domain structures and evolutionary relationships. Sequence alignment was carried out using Prank software (version 14,063) with 1000 iterations (http://wasabiapp.org/software/prank/) [13]. "Codon" model was set for CDS sequences and "AA" model was set for amino acid sequences. A maximum-likelihood tree was inferred using the IQ-TREE software (version 1.3.11) (http://www.iqtree.org/) [14]. A model test was performed using the "-m" option and the best model was auto-selected according to Bayesian information criterion (BIC) score. Bootstrap proportions were obtained using 1000 replications. The tree was visualized with Figtree software (version 1.42, http://tree.bio.ed.ac.uk/software/figtree/).

### Divergence dating of mammalian OASL

Beast software (version 2.47) was employed to determine the timing of diversification between mammalian OASL1 and OASL2 lineages (http://beast.community/) [15]. The Gamma category count was set to 4 and the substitution model was set to "HKY". As for the clock rate and the Yule birth rate, we set the Alpha parameter to 0.001 and the Beta parameter to 1000. Calibrating information from the mouse-rat node (20.90 Mya) was used to calibrate the tree based on our fossil knowledge (http://www.timetree.org/).

### Tertiary structure prediction

Template search was performed using SWISS-MODEL (https://www.swissmodel.expasy.org/) [16]. OAS tertiary structures were predicted by I-TASSER software (https://zhanglab.ccmb.med.umich.edu/I-TASSER/) [17]. The difference between predicted structures and templates was measured by RMSD value. OAS crystal structure was performed and visualized using the Pymol software (version 1.74) with its default options (http://www.pymol.org/).

### Molecular docking

A 19-bp dsRNA was extracted from pig OAS1 structure (PDB: 4RWN). Structures of OAS units were docked with dsRNA or APCPP (an ATP analogue) using the Hex software (version 8.0.0) with correlation type "shape+electro" (http://hex.loria.fr/) [18]. The docking search proceeds by rotating the structures of dsRNA and OAS proteins about their centroids. The initial steric scan was set at *N* = 20, followed by a final search at *N* = 30. Predicted poses with the smallest RMSD value were retained. Pymol software (http://www.pymol.org/) was used to visualize the docking results.

### Positive selection

Positive selection analysis was performed using coding sequences from avian OASL, mammalian OASL1 and OASL2. Sequences were aligned by Prank software (version 140,603, http://wasabiapp.org/software/prank/) in codon model [13]. The aligned sequences were manually trimmed to remove indel or gap areas. Phylogenetic trees were generated by IQ-TREE software (version 1.3.11, http://www.iqtree.org/) [14]. The alignment file and the corresponding tree file representing accepted relationships of species were used as input files. Positive selection analysis was performed by PAML software (version 4.9) [19]. Maximum likelihood-based algorithms were used to calculate ratios of non-synonymous to synonymous substitution rates (d_N_/d_S_). The sites model implemented in PAML calculates d_N_/d_S_ values per site. It then compares models that omit or accommodate elevated d_N_/d_S_. Five models (one ratio, nearly neutral, positive selection, beta, beta and omega) were employed in the positive selection analysis.

## Results

### Ancestral OASs appear to be regulatory factors as opposed to interferon-stimulated genes

**Fig. 1:**
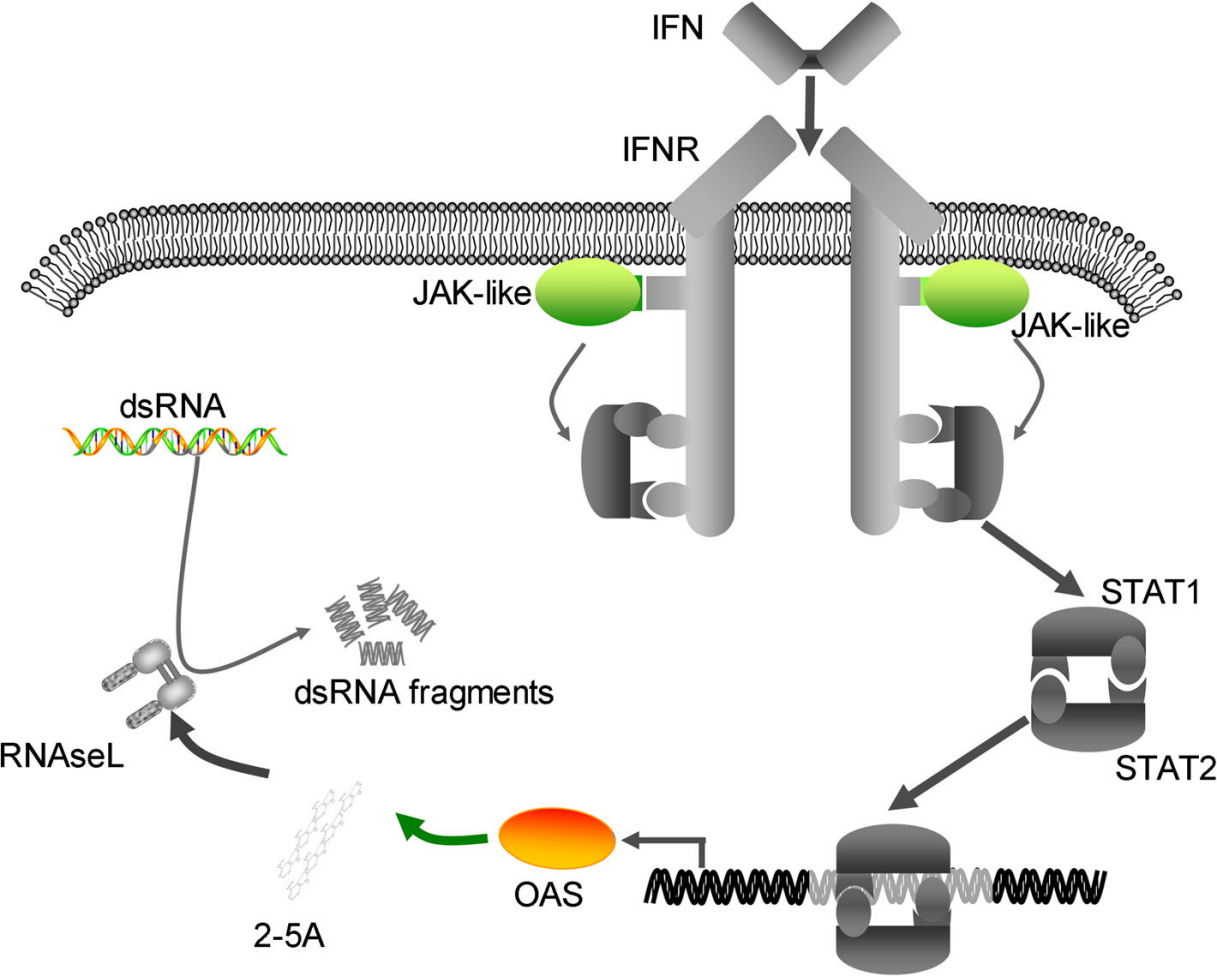
Original OAS genes are not involved in the OAS/RNaseL antiviral pathway. Genes related to the OAS/RNaseL pathway are displayed. IFN, IFNR, STAT1, STAT2, and RNaseL (colored in gray) have not been found in lower Metazoa. OAS genes and JAK-like genes are widely distributed in lower Metazoa

Identification of OASs in sponges, which is a sister-group of the other multi-cellular animals, suggests that OASs exist in ancient organisms [12, 20–22]. To infer whether the OAS/RNaseL pathway in higher animals is also present in lower Metazoa, we searched for seven genes related to the OAS/RNaseL pathway, including OAS, IFN, Interferon receptor (IFNR), Janus kinase (JAK), Signal transducer and activator of transcription 1/2 (STAT1/2) and RNaseL in four sponge species. All four sponges harbored OAS genes and one demosponge (*Amphimedon queenslandica*) contained a fragment of the JAK gene. However, none of them appeared to have IFN, IFNR, STAT1/2, or RNaseL genes (Table 1). This observation is consistent with previous studies, which demonstrate that IFNs exist in fish (such as the rainbow trout, *Oncorhynchus mykiss*), but not in older Metazoa [23]. Similarly, eight lower Metazoa (Acropora coral, *Acropora digitifera*; Starlet sea anemone, *Nematostella vectensis*; Mountainous star coral, *Orbicella faveolata;* Springtails, *Orchesella cincta;* Pacific oyster, *Crassostrea gigas;* Owl limpet, *Lottia gigantea;* Lamp shell, *Lingula anatina;* and Acorn worm, *Saccoglossus kowalevskii*) contain the OAS gene, but do not harbor STAT1/2, IFN, IFNR or RNaseL genes (Table 1). This indicates that the OAS/RNaseL anti-viral pathway does not exist in lower Metazoa (Fig. 1). What's more, the OASL/RIG-I and OASL/IRF7 pathways are also very important in resistance to virus invasion in higher animals. IRF-like genes and RIG-I-like genes seem to exist in lower Metazoa (Additional file 2: dataset 1). Since the UBL domain of the OASL protein plays a key role in the above two pathways, we then predicted the structure of OAS from lower Metazoa (Table 1). None of these OAS proteins had UBL domains. Based on the above observations, we inferred that ancient OASs were not IFN-induced genes involved in the OAS/RNaseL, the OASL/RIG-I or the OASL/IRF7 anti-viral pathways.

**Table 1:** Distribution of OAS related genes in lower Metazoa

Latin name	Common name	OAS	Identity to hOAS1	IFN	STAT 1/2	JAK	IFNR	RNaseL	TOP I	Identity to hTOPI
*Geodia cydonium*	Demosponge	++	26%	–	–	–	–	–	–	
*Tedania ignis*	Fire sponge	++	28%	–	–	–	–	–	–	
*Amphimedon queenslandica*	Demosponge	++	28%	–	–	+	–	–	++	62%
*lubomirskia baicalensis*	Demosponge	++	23%	–	–	–	–	–	–	
*Acropora digitifera*	Acropora coral	++	31%	–	–	+	–	–	++	70%
*Nematostella vectensis*	Startlet sea anemone	++	30%	–	–	+	–	–	++	71%
*Orbicella faveolata*	Mountaius star coral	++	33%	–	–	+	–	–	++	68%
*Orchesella cincta*	Springtails	++	25%	–	–	+	–	–	++	67%
*Crassostrea gigas*	Pacific oyster	++	30%	–	–	+	–	–	++	72%
*Lottia gigantea*	Owl limpet	++	28%	–	–	–	–	–	++	72%
*Lingula anatina*	Lamp shell	++	31%	–	–	+	–	–	++	64%
*Saccoglossus kowalevskii*	Acorn worm	++	28%	–	–	+	–	–	++	73%

++: Nearly complete sequences; +: Fragments; −: No hits found

*hOAS1* human OAS1 protein, *hTOPI* human TOPI protein

**Fig. 2:**
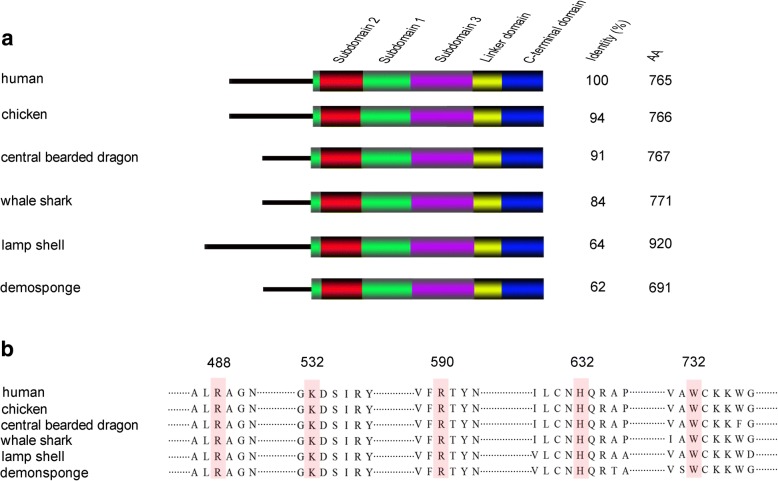
Type I topoisomerase (TOPI) is highly conserved in lower Metazoa. **a** Domains of TOPI. Subdomain 1, subdomain 2, subdomain 3, the linker domain, and C-terminal domain are colored in green, red, purple, yellow and blue. Sequence identity to human TOPI and the size of this protein in the other five species are marked at the right-hand side. **b** Sequence alignment of TOPI proteins from distantly related species. Fragments containing five key residues are displayed and these key residues (colored in pink) show the strict amino acid identity

To infer the possible function of ancient OASs, we focused on the component of their product (2-5A). Exposure of DU145 cells (human, mammals) to physiologic levels of 2-5A results in downregulated expression of TOPI gene by more than two fold [24]. Enzyme activity of calf (mammals) thymus TOPI has been reported to be inhibited by a variety of 2-5A compounds [25]. Our study suggested that 2-5A also downregulated the expression of TOPI gene in HeLa cells (Additional file 1: Figure S1a). Not only in mammals, but also in birds, 2-5A downregulated the expression of TOPI gene (Additional file 1: Figure S1b). It is reasonable to reach the conclusion that regulating TOPI by 2-5A product is an ancient function of OASs and may present in the common ancestor of birds and mammals (tetrapods). These observations remind us that ancestral OASs (such as sponge OAS) may inhibit the expression and activity of TOPI by way of its 2-5A product. Interestingly, we identified TOPI genes in the phylogenetically oldest Metazoa (demosponge) and eight older Metazoa (acropora coral, starlet sea anemone, mountainous star coral, springtails, pacific oyster, owl limpet, lamp shell and acorn worm), which kept OAS genes but lacked three vertebral OAS immune pathways (OAS/RNaseL, OASL/RIG-I and OASL/IRF7). Detailed analysis suggested TOPI proteins of these ancestral Metazoa showed high similarity (> 62%) to that of human and contained conserved domains (core subdomain 1–3, c-terminal domain and Linker domain) (Fig. 2a). Moreover, TOPI proteins from older (i.e. acorn worm and demosponge) to newer (i.e. chicken, human) Metazoa were conserved at five critically functional sites (homologous to Arg488, Lys532, Arg590, His632 and Tyr732 in human TOPI) (Fig. 2b). Such high conservation in domains and protein sequences (especially in critical functional sites) among TOPIs of evolutionally lower and higher animals, together with previous studies implied that ancient OAS might not be an interferon stimulated gene but a regulatory factor for the TOPI gene. However, molecular experiments to further confirm and unravel roles of OASs in Metazoa will be necessary in future studies.

### Factors driving ancestral OASs to be dsRNA sensors

**Fig. 3:**
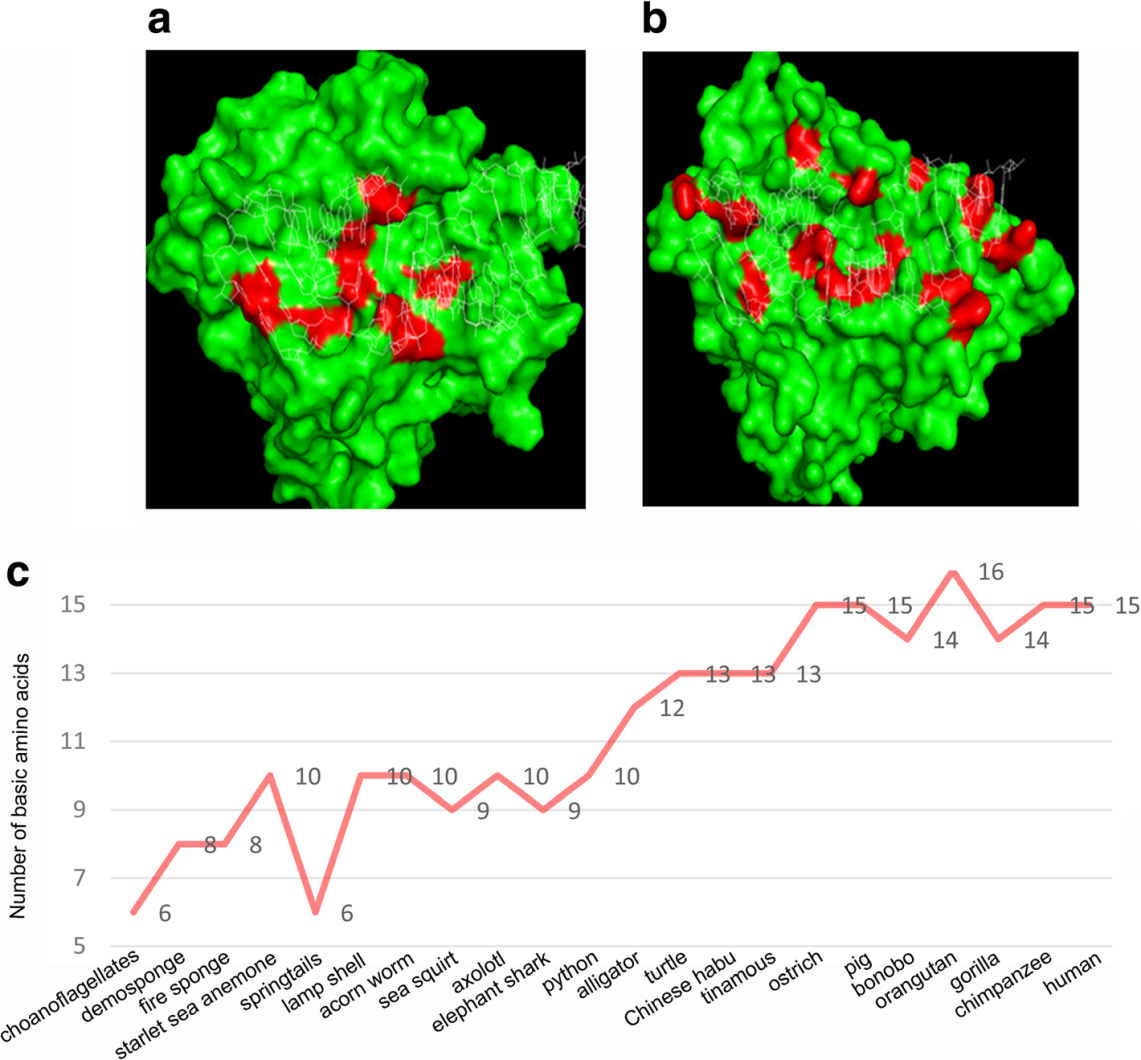
Numbers of basic residues on the OAS/dsRNA interaction interface increase during evolution. **a**, **b** Basic residues on the protein/dsRNA interface of demosponge OAS protein (left) and pig OAS1 protein (right). Basic residues and dsRNA are colored in red and white respectively. **c** Line chart of the number of basic residues on the OAS/dsRNA interface from lower to higher species

After binding dsRNA, mammalian OAS proteins synthesize 2-5A, which activates RNaseL to induce RNA degradation. This is different from the above inferred pattern of ancestral OASs, which seems to produce 2-5A and 3-5A in a dsRNA-independent manner. In order to understand how OASs develop the ability to bind dsRNA, we focused on those positively charged basic residues on the protein/dsRNA interface. Structures of 20 OAS proteins were predicted from evolutionary ancient to modern animals (choanoflagellates, *Monosiga brevicollis*; demosponge; fire sponge, *Tedania ignis*; starlet sea anemone; springtails; lamp shell; acorn worm; sea squirt, *Ciona intestinalis*; axolotl, *Ambystoma mexicanum*; elephant shark, *Callorhinchus millii*; python, *Python bivittatus*; alligator, *Alligator sinensis*; turtle, *Testudines*; Chinese habu, *Protobothrops mucrosquamatus*; tinamous, *Tinamiformes*; ostrich, *Struthio camelus*; bonobo, *Pan paniscus*; orangutan, *Pongo abelii*; gorilla, *Gorilla gorilla*; chimpanzee, *Pan troglodytes*). To determine the accuracy of these predicted structures, we further aligned these predicted OAS structures to two available OAS1 structures (human OAS1 PDB: 4IG8, pig OAS1 PDB: 4RWN). The small RMSD values suggested that these predicted structures shared similar spacial structure with the structures obtained experimentally (Additional file 1: Table S2). OAS protein structures of demosponge and pig with basic residues marked on the protein/dsRNA interface are shown (Fig. 3a-b). The number of basic residues (colored in red) on the dsRNA-binding interface shows a big difference. Further analysis indicated that numbers of basic amino acids on protein/dsRNA interaction interfaces showed a tendency to increase during evolution. One choanoflagellate, one springtail and two sponges (demosponge and fire sponge) have small numbers (6, 6, 8, 8), while one bird (ostrich) and four mammals (pig, chimpanzee, human and orangutan) have large numbers (15, 15, 15, 15, 16) (Fig. 3c). Since dsRNA harbors negatively charged phosphate groups on the surface, we inferred that increasing numbers of positively charged basic residues on the OAS/dsRNA interface in evolutionary modern animals would improve their ability to bind viral dsRNA.

**Fig. 4:**
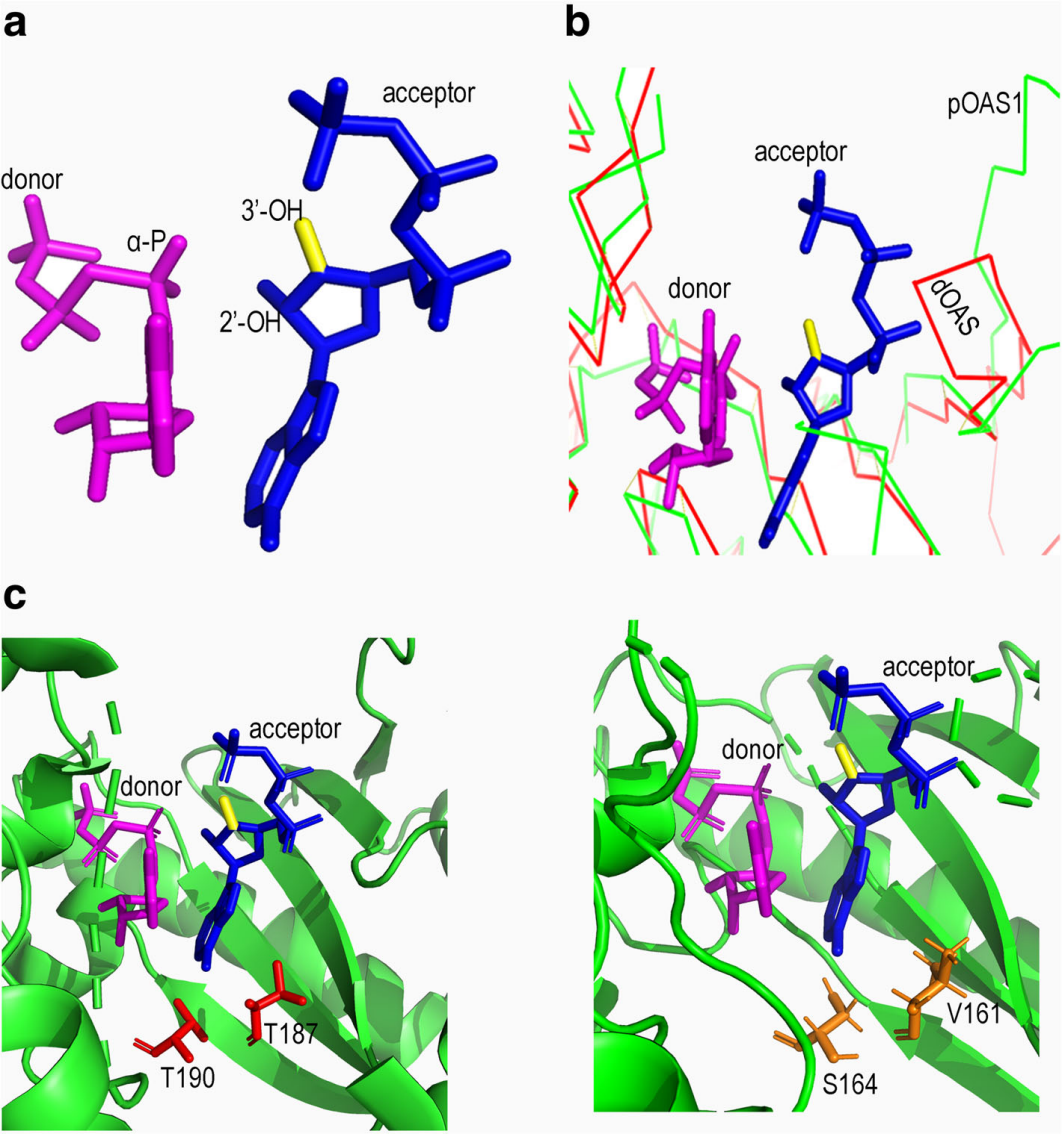
Features of the 2'-specific and 3'-retaining OAS proteins. **a** Conformation of AMP donor and AMP acceptor shared by 2'-specific OAS. In this conformation, the 2'-OH of the AMP acceptor is in close proximity to the alpha-P of the AMP donor. **b** Structure alignment of pig OAS1 (pOAS1, representing 2'-specific OAS) and demosponge OAS (dOAS, representing 3'-retaining OAS). At the right-hand side of the AMP acceptor, pig OAS1 is flat and commodious while demosponge OAS harbors a bulge. **c** Comparative analysis of AMP acceptor pockets. In 2'-specific OAS protein (left), the adenosine base of the AMP acceptor is coordinated both by hydrogen bonds with residues T190 and hydrophobic interactions with T187 (sequence refer to pig OAS1). These two residues help to fix the bottom of the AMP acceptor and stabilize the conformation where the 2'-OH of the AMP acceptor attacks the alpha-P of the AMP donor. However, in 3'-retaining OAS proteins (right), residues being homologous to pig OAS1 T187 and T190 have been mutated. Without interaction of those two residues, the AMP acceptor might move within the pocket, giving both 2'-OH and 3'-OH of AMP acceptor the chance to attack the alpha-P of the AMP donor

OASs in higher Metazoa lost the ability to catalyze the formation of 3'-5'-phosphodiester linkage gradually and became 2'-5'-specific ligases instead. We therefore hypothesized that ancestral OASs should have a conformation that allowed both the 2'-OH and 3'-OH of the AMP acceptor to attack the alpha-P of the AMP donor and synthesized both 2-5A and 3-5A, while modern OASs might have a conformation which allowed only the 2′-OH of the AMP acceptor to attack the alpha-P of the AMP donor and synthesized 2-5A. To infer structural variation that may lead to this change, we took APCPP (an ATP analogue) as substrate and docked it to the tertiary structures of the 2'-specific OAS proteins (i.e. ostrich and human OAS1) and OAS proteins retaining 3'-product synthesis activity (i.e. four sponge OASs). We further compared their structures to the available porcine OAS1·substrate complex structure (2'-specific OAS, PDB: 4RWN) [7]. This effort revealed that 2'-specific OAS proteins showed similar OAS1·substrate complex structure, which was different from those of the four sponge OAS proteins. In general, the 2'-specific OAS proteins had flat and commodious AMP acceptor pockets, and the 2'-OH of the AMP acceptor was close to the alpha-P of the AMP donor. In contrast, the four sponge OASs had a bulge in their AMP acceptor pocket (Fig. 4a, b). The bulge would push the phosphate moiety of the AMP acceptor and rotate the AMP acceptor anti-clockwise in ancestral OASs (i.e. sponge). Such pushing of the bulge seems to move the 3'-OH of the AMP acceptor close to the alpha-P of AMP donor and the 2'-OH move a little further from it. Moreover, structural alignment also highlighted differences in two functional sites between the 2'-specific and sponge OAS proteins. In the former, the adenosine base of the AMP acceptor is coordinated both by hydrogen bonds with residues S186, T190 and Q193 and hydrophobic interactions with residues V78, L149 and T187. What's more, the alpha-phosphate of the AMP acceptor forms of a hydrogen bond with residue R129 (sequence refer to pig OAS1) [7]. However, in the latter, residues being homologous to pig OAS1 T187 and T190 mutated (Fig. 4c). These two residues help to fix the bottom of the AMP acceptor and stabilize the conformation where the 2'-OH of the AMP acceptor attacks the alpha-P of the AMP donor. Without interaction of those two residues, the AMP acceptor might move within the pocket, giving both its 2'-OH and 3'-OH the chance to attack the alpha-P of the AMP donor.

### Phylogeny of the OAS gene family

**Fig. 5:**
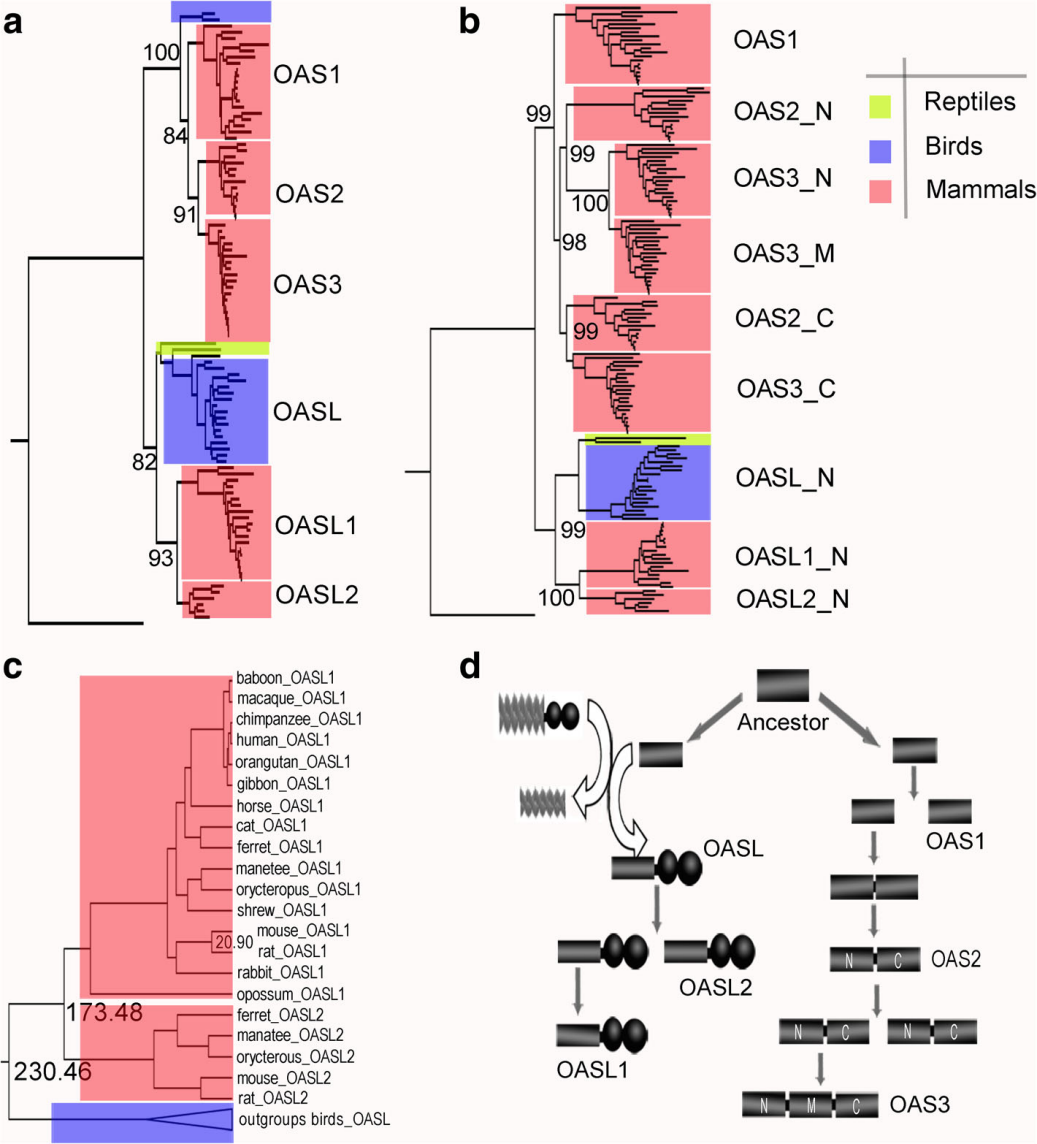
Phylogenetic analysis of the OAS gene family in vertebrates. **a** Maximum-likelihood tree of the OAS gene family based on whole length proteins. **b** Maximum-likelihood tree based on OAS units of OAS family members. OAS2_N and OAS3_N indicate the N terminus of OAS2 and OAS3 proteins. OAS2_C and OAS3_C indicate the C terminus of OAS2 and OAS3 proteins. OAS3_M means the middle domain of the OAS3 protein. **c** Phylogenetic time tree of OASL genes. We calibrated the molecular clock using a divergence time of 20.90 million years (My) for the mouse-rat common ancestor. Two mammalian OASLs diverged about 173.48 million years ago (Mya) and were orthologous to avian OASL. **d** Evolutionary model of the OAS family

To deduce the evolutionary relationship of the existing OAS members, we performed phylogenetic analysis. A Maximum-likelihood (ML) tree was initially constructed using 99 complete or nearly complete OAS protein sequences under the "JTT + F + I + G4" model according to Bayesian information criterion scores (Fig. 5a). This ML tree demonstrates that vertebral OAS genes are grouped into two clusters, namely OAS and OASL. In the OAS subfamily, three members were observed in mammals (OAS1, OAS2, and OAS3). However, OAS1 was found in only two birds (white-throated tinamou and ostrich) from *Palaeognathae* and seem to be lost in *Neognathae* (forty-six *Neognathae* genomes lack OAS1). This was supported by an RNA-seq experiment (unpublished data), which detected two ostrich OAS1 transcripts (Additional file 1: Text S1). Further searching for OAS genes indicated that none of the above 48 avian genomes harbored OAS2 or OAS3 genes (Additional file 1: Table S3). In the OASL subfamily, both birds and reptiles kept one copy of the OASL gene, while mammals retained two copies of OASL (OASL1 and OASL2) genes (Fig. 5a).

To explore the domain coupling and gene duplication scenario of the OAS family, we extracted the core OAS unit to do the following analysis. Multiple sequence alignment was performed using OAS1, N terminus of OAS2, OAS3 and OASL (OAS2_N, OAS3_N, OASL_N, OASL1_N and OASL2_N), C terminus of OAS2 and OAS3 (OAS2_C and OAS3_C), as well as middle domains of OAS3 (OAS3_M) (Fig. 5b). An ML tree was generated under the "HKY + I + G4" model according to Bayesian information criterion scores. The tree shows a major split between OAS1-related domains (OAS1, OAS2_N, OAS2_C, OAS3_N, OAS3_M and OAS3_C) and OASL-related domains (OASL_N, OASL1_N and OASL2_N), which is in accord with the tree topology based on whole-length sequences. In OAS subfamily, domains from OAS2 and OAS3 are divided into two groups. OAS2_C and OAS3_C group together with high bootstrap support, while OAS2_N, OAS3_N and OAS3_M cluster in another group. Within the OASL subfamily, tree topology based on domains is consistent with that based on full-length sequences (Fig. 5b).

In order to understand the evolution model, a controversial issue regarding whether avian OASL and mammalian OASL (OASL1 and OASL2) are orthologues or not must be settled. The divergence time of mammalian OASL genes was calculated using Beast software (version 2.47) [15]. According to this analysis, mammalian OASL1 diverged from OASL2 around 173.48 Mya (Fig. 5c), indicating that the duplication occurred after the time of bird-mammal divergence (312 Mya) but prior to the time of primate-rodent divergence (90 Mya) (http://www.timetree.org/). Therefore, avian OASL genes are orthologous to the ancestral mammalian OASL genes (OASL1 and OASL2).

Based on the above phylogenetic analysis, we proposed the evolutionary pattern of the OAS family: A gene duplication event of OAS and the following domain fusion of UBL give rise to the OASL gene. The ancestor of mammalian OASL was then lineage-specifically duplicated, which resulted in OASL1 and OASL2 (Fig. 5d). In the OAS subfamily, another duplication event resulted in two copies of OAS genes, one copy evolving into OAS1, while the other went through domain coupling and evolved into a two-OAS-domain gene. In a similar manner, the two-domain gene was then duplicated. One copy served as the ancestor of OAS2. The other copy subsequently underwent N-terminal domain coupling, generating the three-OAS-domain gene OAS3 (Fig. 5d).

### Functional diversification of the OAS cluster

**Fig. 6:**
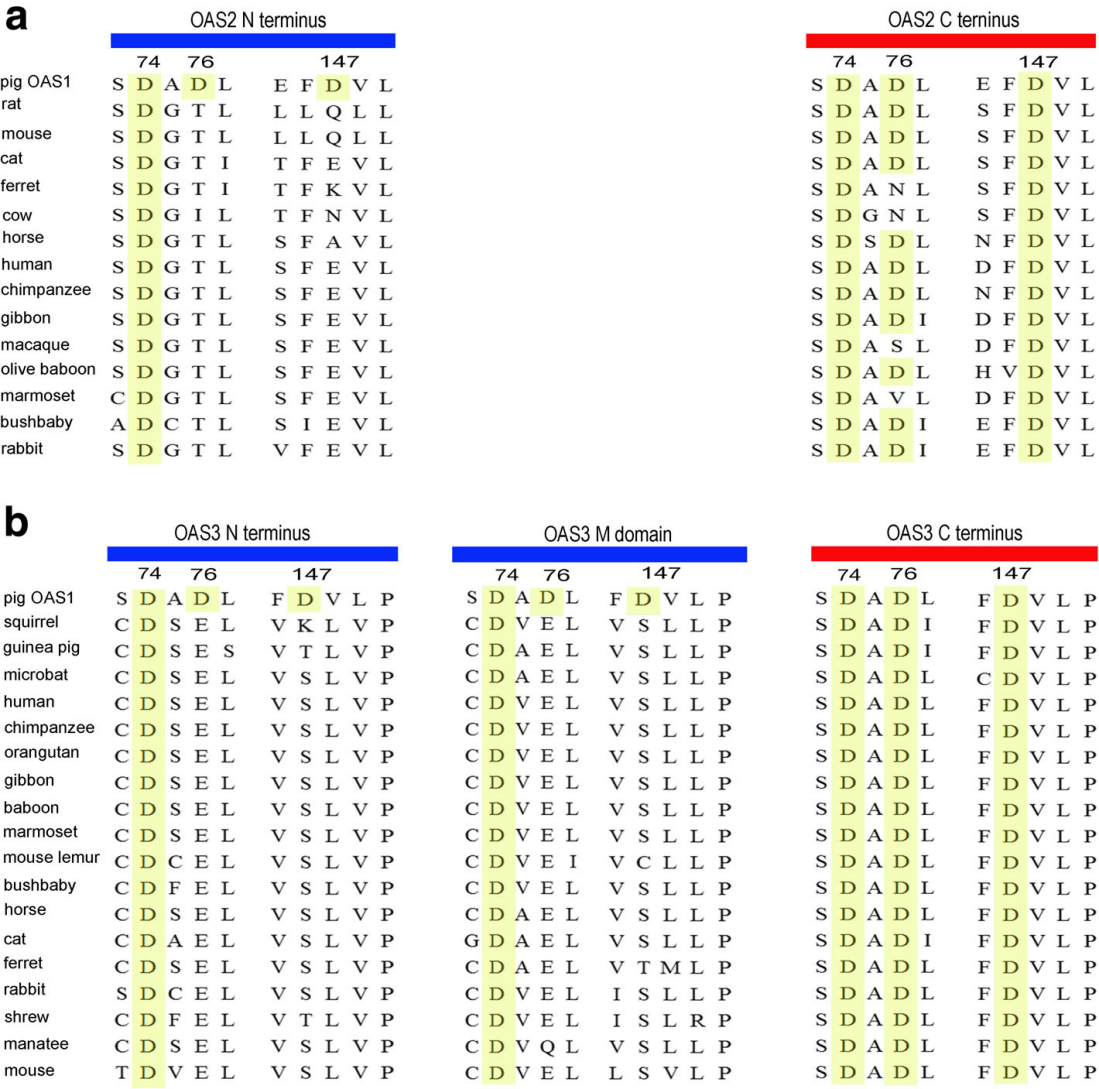
Sequence alignment of OAS2 and OAS3 proteins. Pig OAS1 is set as the reference sequence. Fragments containing triple D residues are demonstrated. Residues homologous to pig OAS1 D74, D76 and D147 are colored in yellow. **a** Sequence alignment of OAS2 N terminus and C terminus. **b** Sequence alignment of OAS3 N terminus, middle domain and C terminus

Since OAS1 binds dsRNA and produces 2-5A with an OAS unit, it appeared that OAS2 and OAS3 contained redundant OAS units. To investigate this hypothesis, we performed a multiple protein sequence alignment using domains from OAS2 (OAS2_N and OAS2_C) and OAS3 (OAS3_N, OAS3_M and OAS3_C). We focused on three conserved Asp (D) sites being critical to the synthesis of 2-5A. This analysis indicated that OAS2_N, OAS3_N and OAS3_M domains showed no OAS activity since they harbored mutations at the active sites. However, most OAS2_C domains and all OAS3_C domains, being highly conserved at these active sites, are enzymatic (Fig. 6a, b). OAS domains from OAS2 and OAS3 have diverged in 2-5A activity, and thus do not seem to be redundant.

We then focused on how OASs diversified their function with active or inactive OAS units. We predicted the structure of OAS2 protein using the I-TASSER software, and found that OAS2 adopted an elongated conformation (Additional file 1: Figure S2). Similarly, the SAXS analysis of OAS3 and the ab-initio and rigid-body models supported that OAS3 adopted an elongated conformation [26]. Since one OAS domain accommodates about 17 bp dsRNA, the linear rearrangement of basic OAS units might facilitate recognizing dsRNA of different lengths. OAS2, which harbors two OAS units, might bind dsRNA more than 34 bp. Likewise, OAS3 possessing three OAS units might prefer to bind dsRNA of more than 51 bp. To validate this hypothesis, we focused on the binding affinity of these domains to dsRNA, including OAS2_N, OAS2_C, OAS3_N and OAS3_C. Structures of these domains was predicted with I-TASSER software and dsRNA was docked to those domains using Hex software (version 8.0.0) [18]. Interestingly, both OAS3_N and OAS3_C showed large energy reduction after docking with dsRNA (Additional file 1: Table S4), supporting the hypothesis that OAS3 might employ the ability to bind long dsRNA. This observation is consistent with the fact that human OAS3 is activated by dsRNA of more than 51 bp in length [27]. However, only OAS2_C, but not OAS2_N had energy decline after docking with dsRNA, which means OAS2_N does not have the ability to bind dsRNA in middle length. Thus, OAS2 might bind short dsRNA (like OAS1), using the OAS2_C domain.

### Functional diversification of the OASL cluster

**Fig. 7:**
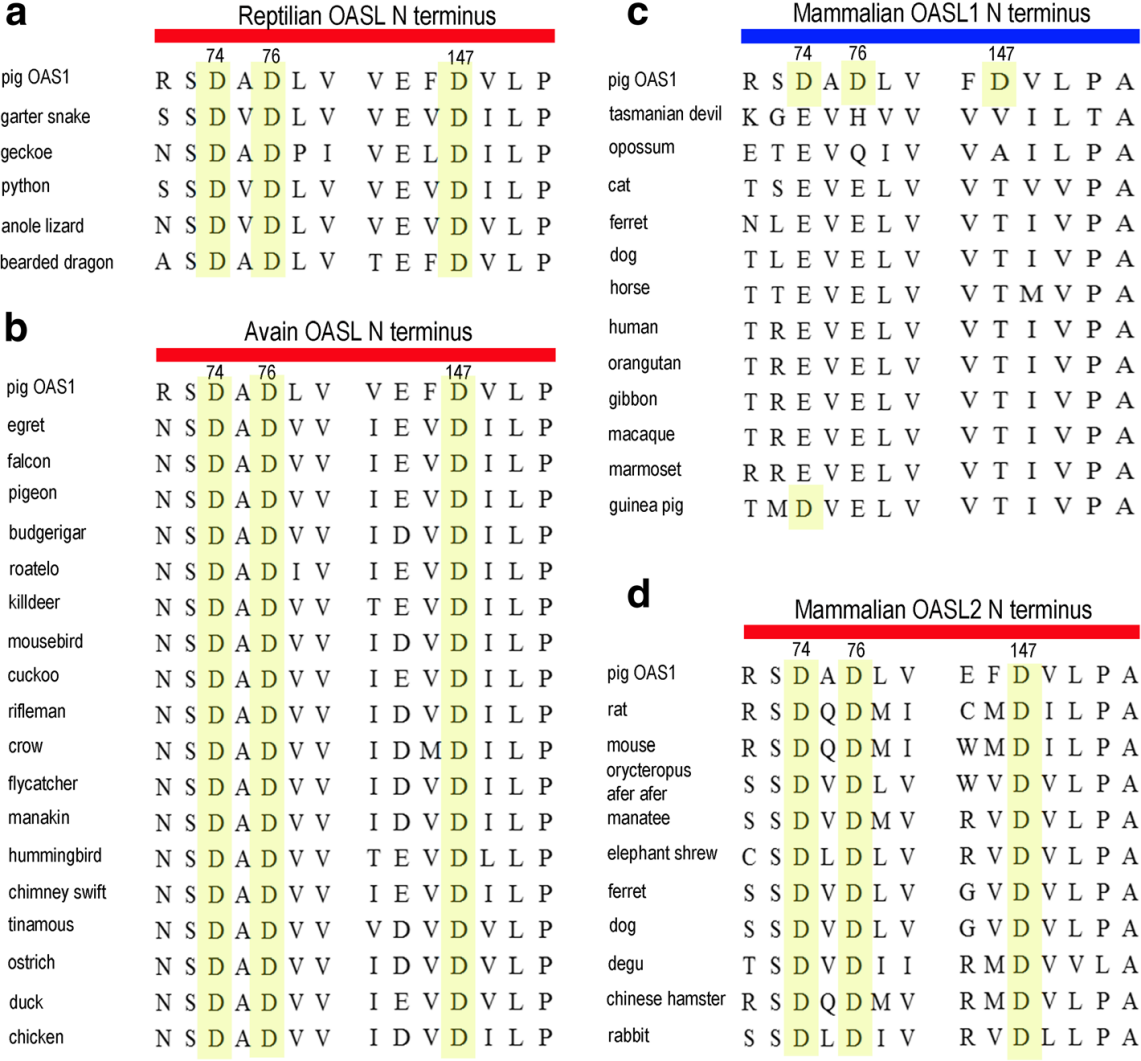
Multiple sequence alignment of OASL proteins. Pig OAS1 protein is set as the reference sequence. Fragments containing triple Asp (D) residues are demonstrated. Residues homologues to pig OAS1 D74, D76 and D147 are colored in yellow. **a** Reptilian OASL. **b** Avian OASL. **c** Mammalian OASL1. **d** Mammalian OASL2

Phylogenetic analysis indicated that reptiles and birds had one OASL gene, while mammalian lineage-specific duplication resulted in two OASL members (OASL1 and OASL2). Probably due to the gene duplication force, mammalian OASL1 (d_N_/d_S_ = 0.337) and OASL2 (d_N_/d_S_ = 0.366) were under stronger positive selection pressure than avian OASL (d_N_/d_S_ = 0.294). We further identified eight, two, and ten positive selection sites in avian OASL, mammalian OASL1, and mammalian OASL2, respectively (Additional file 1: Table S5 and Figure S3). Such a difference in evolutionary pressure might contribute to functional diversification of tetrapod OASLs. We then focused on three conserved Asp (D) residues, which are critical to the enzymatic activity. We performed sequence alignment of the OASL subfamily to identify mutations at these sites. Three D sites in reptilian and avian OASLs were conserved (Fig. 7a-b), indicating that these OASLs were enzymatic. This was further supported by our recent study, where we showed that duck OASL and ostrich OASL exhibit catalytic activity [28]. In mammalian OASLs, mutations at three D sites drove their functional diversification, where OASL1 was non-enzymatic partly due to mutations of these conserved D sites (Fig. 7c). In contrast, OASL2 retained three conserved catalytic D residues and showed 2-5A enzyme activity (Fig. 7d). We then focused on enzymatic OASL genes, including avian OASL and mammalian OASL2. Interestingly, avian OASL genes evolved at a lower rate when compared to that of mammalian OASL2, partly due to the fact that the latter was driven by the evolutionary force of gene duplication (Additional file 1: Figure S4). Further analysis indicated that mutations in the UBL domain of tetrapod OASL might contribute to their functional diversity. *Sauropsida* (reptiles and birds) OASL genes contain more basic amino acids in the second UBL domain, while mammalian OASL2 genes harbor more basic residues in the first UBL domain (Additional file 1: Figure S5). These observations are in agreement with the hypothesis that the UBL domain of *sauropsida* OASLs might execute anti-viral activity in a pattern which is different from that of mammalian OASL2.

## Discussion

Gene duplication produces two or more copies of a particular gene and provides a major genetic resource to evolve phenotypic complexity. Driven by purifying selection, many superfluous duplicates are lost or become pseudo-genes. However, some are preserved due to the fact that they increase gene dosage, acquire a novel function, or retain part function of the ancestral gene. Such neo-functionalization, sub-functionalization and specialization result in two copies being functionally distinct from each other, thus making a great challenge for predicting gene functions according to comparative functional studies in model species [29]. For example, mammalian and avian defensins are short cationic peptides and contribute to the immune response to viral infection [30, 31]. However, the homologs of avian and mammalian defensins in platypus and reptiles were subsequently diversified to venom [32]. Here, we found that the OAS family were distributed widely among Metazoa. We inferred ancient OASs were not IFN-induced genes involved in the OAS/RNaseL immune response like avian and mammalian homologs since they lacked both upstream and downstream genes. Instead, ancestral OASs seemed to act as regulatory factors affecting gene expression.

The arms race between host and pathogen results in significant expansion and adaptive evolution of genes involved in the immune response [33]. The OAS family represented such a scenario, where they have developed anti-viral activity in Metazoa. On the one hand, evolutionary selection increased the number of basic residues on OAS/dsRNA interaction interfaces, which in return improved their binding affinity to viral dsRNA. On the other hand, higher Metazoa optimized the structure of the AMP acceptor pocket, which allowed only the 2’-OH of the AMP acceptor to attack the alpha-P of the AMP donor. Moreover, OASs diversified through lineage-specific gene duplication deletion or adaptive evolution. Such difference in evolutionary pattern contributed to component and functional diversity of the OAS repertoires, where most birds hold only one conserved OAS member (OASL). In contrast, the mammalian OAS family is more complex. The human genome harbors four OAS members: OAS1, OAS2, OAS3 and OASL1. Human OAS1 and OAS2 have proved to be ineffective for activation of RNaseL upon virus infection [28]. Human OASL1 lost OAS enzyme activity by mutation at three D residues. In fact, only OAS3 in the human OAS family can initiate the OAS/RNaseL pathway upon viral infection [6]. In the mouse genome, although twelve OAS genes are found, six OAS1 deviate OAS enzyme activity due to mutation at three D sites. Moreover, it has been reported that mouse OASL1 not only lost OAS enzyme activity but also inhibited the translation of IRF7 by binding to its 5’-UTR. Such diversification would effectively reduce redundant functions, negatively regulate IFN signalling and prevent hosts from a hyper-inflammatory response.

Evolutionary analysis has shed light on the functional study of these genes. Mutations of three conserved D residues suggested that some newly duplicated OAS domains or genes lost OAS enzyme activity and might get new functions. This hypothesis was supported by recent studies, which indicated that human OAS2 downregulated the expression of T-cell receptor CD-ξ chain via caspase-3 activation in oral cancer [34]. Moreover, mouse OAS2 was found to be involved in mammary development and lactation [35]. However, knowledge regarding functions of those OAS members is still limited. Therefore, functional analysis of new OAS members using genetic manipulations will be meaningful to further explore the diversification of the OAS repertoires.

## Conclusions

Evolution of the OAS gene family presents a scenario where we show that immune genes can be developed from non-immune genes and further diversify their anti-viral activity. Ancient OAS generated both 2-5A and 3-5A products in a dsRNA independent manner. Driven by evolutionary force (such as positive selection), higher metazoan OASs increased their dsRNA binding affinity and became 2-5A specific. Higher metazoa further diversified their repertoires through gene duplication, domain coupling or gene fusion. In return, functional redundancy of OAS duplications accelerated evolutionary rate and adaptive selection, which resulted in deletion, neo-functionalization or sub-functionalization of OASs in higher Metazoa. These phylogenetic results will provide insight into functional studies of OASs in metazoa.

## Additional files

Additional file 1: **Text S1.** Two transcripts of ostrich OAS1. **Table S1.** Primers used for real time quantitative RT-PCR. **Table S2.** Information on OAS structures. **Table S3.** Distribution of avian OAS genes. **Table S4.** Energy change of OAS domains after docking with dsRNA. **Table S5.** Positive selection analysis of the OASL subfamily. **Figure S1.** 2-5A downregulated the expression of TOP I gene. Cells were transfected with 2-5A in different concentration. TOP I was detected at 6 h post transfection. (a) HeLa cells. (b) DF1 cells. **Figure S2.** Predicted structure of human OAS2 protein. N terminus (left) and C terminus (right) of OAS2 were colored in red and green respectively. **Figure S3.** Distribution of positively selected sites for the OASL gene. Sequence alignment was carried out using the PRANK software (version 140,603) and positively selected sites were predicted using PAML software (version 4.9). Sites displaying significant ω values were shown. Sequences for avian OASL, mammalian OASL1 and OASL2 refer to ostrich OASL, alpaca OASL1 and shrew OASL2, respectively. **Figure S4.** Evolutionary rate of mammalian OASL2 and avian OASL genes. Sequence alignment was performed by PRANK software (version 140,603) and evolutionary rate was calculated using BEAST software (version 2.47). (a) Mammalian OASL2. (b) Avian OASL. It is clear that mammalian OASL2 has evolved faster than avian OASL. **Figure S5.** Isoelectric point of residues in two UBL domains of OASLs (C terminus of OASL protein; ~ 150 residues). (a-c) Reptilian and avian UBL domains. Common garter snake, duck and chicken OASLs were selected as reptilian and avian OASL representatives. (d-e) Mammalian UBL domains. Mouse and rat OASL2 were selected to represent mammalian OASL2. Avian and reptilian OASL proteins contain more basic residues (pink box) in the second UBL domain, while mammalian OASL proteins prefer to harbor basic residues (pink box) in the first UBL domain. (DOCX 2920 kb)

Additional file 2: Sequences used in this study. (TXT 296 kb)

## Abbreviations

_N/_M/_C: N terminal / middle / C terminal domain of
2-5A/3-5A: 2′-5′-linked oligoadenylate/3′-5′-linked oligoadenylate
dsRNA: Double-stranded RNA
IRF7: Interferon regulatory factor 7
ML: Maximum likelihood
OAS: /OASL Oligoadenylate synthetase/Oligoadenylate synthetase like
PAP: Poly adenosine polymerases
RIG-I: Retinoic acid-inducible gene I
RNaseL: Ribonuclease L
TOPI: Type I topoisomerase
UBL: Ubiquitin-like domain
