# Supplementary material for: Origin and development of oligoadenylate synthetase immune system
Source: BMC Evol Biol. 2018 Dec 27;18:201. doi: 10.1186/s12862-018-1315-x (PMC6307210; doi:10.1186/s12862-018-1315-x)
Supplement: Supplementary file 1 — Text S1. Two transcripts of ostrich OAS1. Table S1. Primers used for real time quantitative RT-PCR. Table S2. Information on OAS structures. Table S3. Distribution of avian OAS genes. Table S4. Energy change of OAS domains after docking with dsRNA. Table S5. Positive selection analysis of the OASL subfamily. Figure S1. 2-5A downregulated the expression of TOP I gene. Cells were transfected with 2-5A in different concentration. TOP I was detected at 6 h post transfection. (a) HeLa cells. (b) DF1 cells. Figure S2. Predicted structure of human OAS2 protein. N terminus (left) and C terminus (right) of OAS2 were colored in red and green respectively. Figure S3. Distribution of positively selected sites for the OASL gene. Sequence alignment was carried out using the PRANK software (version 140,603) and positively selected sites were predicted using PAML software (version 4.9). Sites displaying significant ω values were shown. Sequences for avian OASL, mammalian OASL1 and OASL2 refer to ostrich OASL, alpaca OASL1 and shrew OASL2, respectively. Figure S4. Evolutionary rate of mammalian OASL2 and avian OASL genes. Sequence alignment was performed by PRANK software (version 140,603) and evolutionary rate was calculated using BEAST software (version 2.47). (a) Mammalian OASL2. (b) Avian OASL. It is clear that mammalian OASL2 has evolved faster than avian OASL. Figure S5. Isoelectric point of residues in two UBL domains of OASLs (C terminus of OASL protein; ~ 150 residues). (a-c) Reptilian and avian UBL domains. Common garter snake, duck and chicken OASLs were selected as reptilian and avian OASL representatives. (d-e) Mammalian UBL domains. Mouse and rat OASL2 were selected to represent mammalian OASL2. Avian and reptilian OASL proteins contain more basic residues (pink box) in the second UBL domain, while mammalian OASL proteins prefer to harbor basic residues (pink box) in the first UBL domain. (DOCX 2920 kb) [file 12862_2018_1315_MOESM1_ESM.docx]

**Additional file 1**

**Text S1. Two transcripts of ostrich OAS1**

>transcript_1

atgctgtcacagctgctgtcgtgcctcatggagctctacaacaccccgtcagagcagctg

gacaagttcatctacgaggtgctgcagcctgaccgcacattcctggagcagctgcgctgt

gctgtgcacaccatctgcgagttcctgcgggacaactgctttgcgggggctcctccaccc

cgaacacgggtgcttaaggtcgtgaagggtggctctgcaggcaaaggcacggccctcaaa

aagagctctgacgctgacctcgtggtgtttctcagctgctttgaggactataaggaccag

gagaaaaaccgggcagagatcatccgtgagatccagaagaggctggtggagtgccagcag

cagaagcattttgaggtggagtttgaagtgagtcgttggtcaaatccccgcgtgctgagc

ttccagctgagctccaagacgctccatgagtccatcagcttcgatgtgctgccggcctat

gacgcgctacaccaccttgtctctggctacaaggtcaaccccacagtttacatccagctc

ttccagcagtgctcgcggggaggtgaattctccacctgcttcacggagctgcaacgggac

ttcatcatcagccgccccaccaaagtgaagagcctgattcgcctggtgaagcactggtac

aagacgtacatctgtccacacaagtgggcgctgagaggtggcgagaccctgccaccgcag

tatgccctggagctcctgacagtctacgcctgggagcaggggagtggggaaacaaacttc

agtgtggccaaggccttccgcacagtgctggagctgctccagcactaccagcagctctgt

gtctactggactgtcaactacgacttcagggatgcgactctgagttgccacctgtcatcc

cagctcagcaaaagcaggcctgtcatcctcgacccggctgacccaaccaatattgtgggc

aaagggagccgctgggacctggtggcgaaggaggctgaaaagtgctgtagtcagcgatgc

tgcatgtacagcaatggagtgcctgtgcaaccatgggacgtgtcgcctgagcaaacacgc

tga

>transcript_2 (premature termination）

atgctgtcacagctgctgtcgtgcctcatggagctctacaacaccccgtcagagcagctg

gacaagttcatctacgaggtgctgcagcctgaccgcacattcctggagcagctgcgctgt

gctgtgcacaccatctgcgagttcctgcgggacaactgctttgcgggggctcctccaccc

cgaacacgggtgcttaaggtcgtgaagggtggctctgcaggcaaaggcacggccctcaaa

aagagctctgacgctgacctcgtggtgtttctcagctgctttgaggactataaggaccag

gagaaaaaccgggcagagatcatccgtgagatccagaagaggctggtggagtgccagcag

cagaagcattttgaggtggagtttgaagtgagtcgttggtcaaatccccgcgtgctgagc

ttccagctgagctccaagacgctccatgagtccatcagcttcgatgtgctgccggcctat

gacgcgctacgtacggtctgctgccctgcttggctgcccactctgctgggcgtttcctga

gccatgggaccctcctgctcagcctggcccttgcctacagcaggacttgctggggctgaa

gtgtcccagctccctgtccctgtcgtgcagccttcacatcggtatctttctgttttgctt

ctccctcgcagaccaccttgtctctggctacaaggtcaaccccacagtttacatccagct

cttccagcagtgctcgcggggaggtgaattctccacctgcttcacggagctgcaacggga

cttcatcatcagccgccccaccaaagtgaagagcctgattcgcctggtgaagcactggta

caagacgtacatctgtccacacaagtgggcgctgagaggtggcgagaccctgccaccgca

gtatgccctggagctcctgacagtctacgcctgggagcaggggagtggggaaacaaactt

cagtgtggccaaggccttccgcacagtgctggagctgctccagcactaccagcagctctg

tgtctactggactgtcaactacgacttcagggatgcgactctgagttgccacctgtcatc

ccagctcagcaaaagcaggcctgtcatcctcgacccggctgacccaaccaatattgtggg

caaagggagccgctgggacctggtggcgaaggaggctgaaaagtgctgtagtcagcgatg

ctgcatgtacagcaatggagtgcctgtgcaaccatgggacgtgtcgcctgagcaaacacg

ctga

**Table S1.** Primers used for real time quantitative RT-PCR

| Gene | Primer sequences (5′-3′) |
| --- | --- |
| Chicken TOP1 | AGCAGCCTGAGGATGACC |
|  | CAAGCAGCCTGAGGATGAC |
| Chicken GAPDH | TGCCCAGAACATCATCCCA |
|  | CGGCAGGTCAGGTCAACAA |
| Human TOP1 | TCGAAGCGGATTTCCGATTGA |
|  | CTTTGTGCCGGTGTTCTCGAT |
| Human GAPDH | CTGGGCTACACTGAGCACC |
|  | AAGTGGTCGTTGAGGGCAATG |

**Table S2.** Information on OAS structures

| Species | Source | Template | Identity | Coverage | Estimated RMSD |
| --- | --- | --- | --- | --- | --- |
| Choanoflagellates | predicted | 4RWN:A | 0.33 | 0.99 | 4.6±3.0Å |
| Domosponge | predicted | 4RWN:A | 0.28 | 0.97 | 4.2±2.8Å |
| Fire sponge | predicted | 4RWN:A | 0.28 | 0.98 | 3.9±2.6Å |
| Starlet sea  anemone | predicted | 4RWN:A | 0.30 | 0.99 | 3.4±2.4Å |
| Springtails | predicted | 4RWN:A | 0.25 | 0.91 | 5.6±3.5Å |
| Lamp shell | predicted | 4RWN:A | 0.31 | 0.96 | 4.2±2.8Å |
| Acorn worm | predicted | 4RWN:A | 0.28 | 0.96 | 4.5±2.9Å |
| Sea squirt | predicted | 4RWN:A | 0.29 | 0.90 | 8.1±4.4Å |
| Axolotl | predicted | 4RWN:A | 0.45 | 0.87 | 6.1±3.8Å |
| Elephant shark | predicted | 4RWN:A | 0.29 | 0.95 | 4.0±2.7Å |
| Python | predicted | 4RWN:A | 0.48 | 0.96 | 3.9±2.6Å |
| Alligator | predicted | 4RWN:A | 0.55 | 0.98 | 3.4±2.4Å |
| Turtle | predicted | 4RWN:A | 0.51 | 0.97 | 3.6±2.5Å |
| Chinese habu | predicted | 4RWN:A | 0.53 | 0.99 | 3.3±2.3Å |
| Tinamous | predicted | 4RWN:A | 0.54 | 0.99 | 3.3±2.3Å |
| Osrich | predicted | 4RWN:A | 0.52 | 0.94 | 4.2±2.8Å |
| Pig | PDB database |  |  |  |  |
| Bonobo | predicted | 4IG8:A | 0.95 | 0.97 | 3.4±2.3Å |
| Orangutan | predicted | 4IG8:A | 0.93 | 0.98 | 3.4±2.4Å |
| Gorilla | predicted | 4IG8:A | 0.96 | 0.98 | 3.4±2.4Å |
| Chimpanzee | predicted | 4IG8:A | 0.93 | 0.97 | 3.3±2.3Å |
| Human | PDB database |  |  |  |  |

**Table S3.** Distribution of avian OAS genes

| Latin name | Common name | Gene ID | Strand | Transcript length | Number of exons | Number of residues |
| --- | --- | --- | --- | --- | --- | --- |
| *Acanthisitta chloris* | rifleman | [103811553](http://www.ncbi.nlm.nih.gov/gene/103811553?report=gene_table) | forward | 1572 bp | 6 | 523 aa |
| *Anas platyrhynchos* | mallard | 101800895 | forward | 1512 bp | 6 | 503 aa |
| *Apaloderma vittatum* | bar-tailed trogon | 104269822 | forward | 1519 bp | 6 | 505 aa |
| *Aptenodytes forsteri* | emperor penguin | 103895244 | forward | 1626 bp | 7 | 545 aa |
| *Balearica regulorum gibbericeps* | grey crowned-crane | 104629345 | forward | 1500 bp | 6 | 499 aa |
| *Buceros rhinoceros silvestris* | rhinoceros hornbill | 104499359 | forward | 1402 bp | 7 | 466 aa |
| *Calypte anna* | anna's hummingbird | 103533238 | reverse | 1455 bp | 7 | 484 aa |
| *Caprimulgus carolinensis* | chuck-will's-widow | 104526707 | reverse | 1302 bp | 5 | 433 aa |
| *Cariama cristata* | red-legged seriema | 104165675 | forward | 1314 bp | 5 | 437 aa |
| *Chaetura pelagica* | chimney swift | 104387124 | forward | 1587 bp | 7 | 528 aa |
| *Charadrius vociferus* | killdeer | 104282965 | reverse | 1503 bp | 7 | 500 aa |
| *Chlamydotis macqueenii* | macqueen's bustard | 104483817 | forward | 561 bp | 3 | 186 aa |
| *Colius striatus* | speckled mousebird | 104550038 | forward | 1530 bp | 6 | 509 aa |
| *Columba livia* | rock pigeon | 102086122 | reverse | 1682 bp | 6 | 518 aa |
| *Corvus brachyrhynchos* | american crow | 103614386 | reverse | 1710 bp | 7 | 569 aa |
| *Cuculus canorus* | common cuckoo | 104066750 | reverse | 1557 bp | 6 | 518 aa |
| *Egretta garzetta* | little egret | 104126663 | forward | 1560 bp | 6 | 519 aa |
| *Eurypyga helias* | sunbittern | 104517116 | reverse | 1383 bp | 7 | 460 aa |
| *Falco cherrug* | saker falcon | 102050726 | forward | 1545 bp | 6 | 514 aa |
| *Falco peregrinus* | peregrine falcon | 101910501 | reverse | 1545 bp | 6 | 514 aa |
| *Ficedula albicollis* | collared flycatcher | [101807457](http://asia.ensembl.org/Ficedula_albicollis/Gene/Summary?db=core;g=ENSFALG00000008796;r=JH603227.1:4159864-4162340;t=ENSFALT00000009215) | forward | 1989 bp | 6 | 536 aa |
| *Fulmarus glacialis* | northern fulmar | 104075318 | reverse | 1068 bp | 6 | 355 aa |
| *Gallus gallus* | chicken | [395908](http://asia.ensembl.org/Gallus_gallus/Gene/Summary?db=core;g=ENSGALG00000013723;r=12:3226270-3228218) | reverse | 1546 bp | 6 | 514 aa |
| *Gavia stellata* | red-throated loon | 104255679 | forward | 946 bp | 6 | 314 aa |
| *Geospiza fortis* | medium ground-finch | 102035058 | reverse | 1770 bp | 7 | 589 aa |
| *Leptosomus discolor* | cuckoo roller | 104340299 | reverse | 1569 bp | 6 | 531 aa |
| *Manacus vitellinus* | golden-collared manakin | 103755321 | reverse | 1575 bp | 6 | 524 aa |
| *Meleagris gallopavo* | turkey | 104913141 | reverse | 1571 bp | 6 | 496 aa |
| *Melopsittacus undulatus* | budgerigar | 101870487 | reverse | 1557 bp | 6 | 518 aa |
| *Merops nubicus* | carmine bee-eater | 103776598 | forward | 1395 bp | 7 | 464 aa |
| *Mesitornis unicolor* | brown roatelo | 104538747 | forward | 1572 bp | 6 | 523 aa |
| *Nestor notabilis* | kea | 104405990 | reverse | 1362 bp | 5 | 452 aa |
| *Nipponia nippon* | crested ibis | 104008725 | forward | 1545 bp | 6 | 514 aa |
| *Opisthocomus hoazin* | hoatzin | 104336385 | forward | 749 bp | 3 | 248 aa |
| *Pelecanus crispus* | dalmatian pelican | 104026856 | forward | 1399 bp | 6 | 465 aa |
| *Phaethon lepturus* | white-tailed tropicbird | 104617225 | forward | 1346 bp | 7 | 447 aa |
| *Phalacrocorax carbo* | great cormorant | 104047741 | forward | 1542 bp | 6 | 513 aa |
| *Picoides pubescens* | downy woodpecker | 104306686 | forward | 1650 bp | 7 | 549 aa |
| *Pseudopodoces humilis* | tibetan ground-tit | 102099265 | forward | 1791 bp | 9 | 596 aa |
| *Pterocles gutturalis* | yellow-throated sandgrouse | 104467401 | forward | 889 bp | 6 | 295 aa |
| *Pygoscelis adeliae* | adelie penguin | 103923313 | reverse | 510 bp | 3 | 169 aa |
| *Serinus canaria* | common canary | 103824282 | forward | 1728 bp | 6 | 515 aa |
| *Struthio camelus australis* | ostrich | 104142894 | reverse | 1640 bp | 6 | 529 aa |
|  |  | 104140738 | reverse | 1083 bp | 6 | 360 aa |
| *Taeniopygia guttata* | zebra finch | 100224927 | reverse | 1500 bp | 7 | 500 aa |
| *Tauraco erythrolophus* | red-crested turaco | 104376549 | forward | 1419 bp | 7 | 472 aa |
| *Tinamus guttatus* | white-throated tinamou | 104566708 | forward | 1335 bp | 7 | 444 aa |
|  |  | 104577834 | reverse | 1563 bp | 6 | 515 aa |
| *Tyto alba* | barn owl | 104358453 | reverse | 1279 bp | 5 | 425 aa |
| *Zonotrichia albicollis* | white-throated sparrow | 102064521 | forward | 1564 bp | 7 | 487 aa |

**Table S4.** Energy change of OAS domains after docking with dsRNA

| Domain | Ligand (19bp) | Energy score | RSM score |
| --- | --- | --- | --- |
| Human OAS3 N terminus | dsRNA | -2299.0 | 2.97 |
| Human OAS3 C terminus | dsRNA | -1421.9 | 2.89 |
| Chimpanzee OAS3 N terminus | dsRNA | -2299.0 | 2.97 |
| Chimpanzee OAS3 C terminus | dsRNA | -1421.9 | 2.89 |
| Mouse OAS3 N terminus | dsRNA | -1486.4 | 0.85 |
| Mouse OAS3 C terminus | dsRNA | -1655.1 | 2.96 |
| Cat OAS3 N terminus | dsRNA | -1860.5 | 2.03 |
| Cat OAS3 C terminus | dsRNA | -1983.6 | 2.52 |
| Horse OAS3 N terminus | dsRNA | -713.9 | 2.28 |
| Horse OAS3 C terminus | dsRNA | -849.4 | 2.01 |
| Walrus OAS3 N terminus | dsRNA | -1621.5 | 2.51 |
| Walrus OAS3 C terminus | dsRNA | -1278.7 | 2.91 |
| Human OAS2 N terminus | dsRNA | 462.1 | 2.63 |
| Human OAS2 C terminus | dsRNA | -2026.4 | 2.90 |
| Mouse OAS2 N terminus | dsRNA | 169.8 | 2.64 |
| Mouse OAS2 C terminus | dsRNA | -1571.1 | 2.71 |

**Table S5.** Positive selection analysis of the OASL subfamily

| Gene | N | M0 | M1:M2 | M7:M8 | M8 | |
| --- | --- | --- | --- | --- | --- | --- |
|  |  |  |  |  | Site | P value |
| Avian OASL | 16 | Kappa  (ts/tv)  = 3.565  Omega (dN/dS)  = 0.294 | lnL(M1)=  -12204.256  lnL(M2)=  -12176.371  LRT=  55.778  P=  7.726e-13 | lnL(M7)=  -12144.481  lnL(M8)=  -12116.656  LRT=  55.650  P=  8.237e-13 | 89 E | 0.999 |
|  |  |  |  |  | 99 E | 0.959 |
|  |  |  |  |  | 207 D | 0.988 |
|  |  |  |  |  | 315 T | 0.974 |
|  |  |  |  |  | 316 S | 0.999 |
|  |  |  |  |  | 336 V | 0.977 |
|  |  |  |  |  | 364 L | 0.981 |
|  |  |  |  |  | 373 P | 0.957 |
| Mammalian OASL1 | 37 | Kappa  (ts/tv)  =3.526  Omega (dN/dS)  =0.337 | lnL(M1)=  -15801.919  lnL(M2)=  -15793.697  LRT=  16.444  P=  2.687e-4 | lnL(M7)=  -15777.498  lnL(M8)=  -15760.556  LRT=  33.884  P=  4.387e-8 | 104 Y | 0.979 |
|  |  |  |  |  | 154 H | 0.989 |
| Mammalian OASL2 | 31 | Kappa  (ts/tv)  =3.022  Omega (dN/dS)  =0.366 | lnL(M1)=  -17735.080  lnL(M2)=  -17684.849  LRT=  100.462  P=  0.000e0 | lnL(M7)=  -17693.565  lnL(M8)=  -17633.815  LRT=  119.500  P=  0.000e0 | 4 R | 0.991 |
|  |  |  |  |  | 96 D | 0.999 |
|  |  |  |  |  | 100 I | 1.000 |
|  |  |  |  |  | 171 E | 0.991 |
|  |  |  |  |  | 212 H | 0.996 |
|  |  |  |  |  | 328 A | 0.953 |
|  |  |  |  |  | 364 K | 0.982 |
|  |  |  |  |  | 368 T | 1.000 |
|  |  |  |  |  | 435 S | 1.000 |
|  |  |  |  |  | 448 P | 0.998 |

Positively selective analysis were predicted with the PAML software version 4.9 Codeml package.

N: number of species

M0: model 0, one ratio

M1: model 1, nearly neutral (2 categories)

M2: model 2, positive selection (3 categories)

M7: model 7, beta

M8: model 8, beta and omega

Sequences for avian OASL, mammalian OASL1, and mammalian OASL2 were referred to ostrich OASL, alpaca OASL1 and shrew OASL2, respectively.


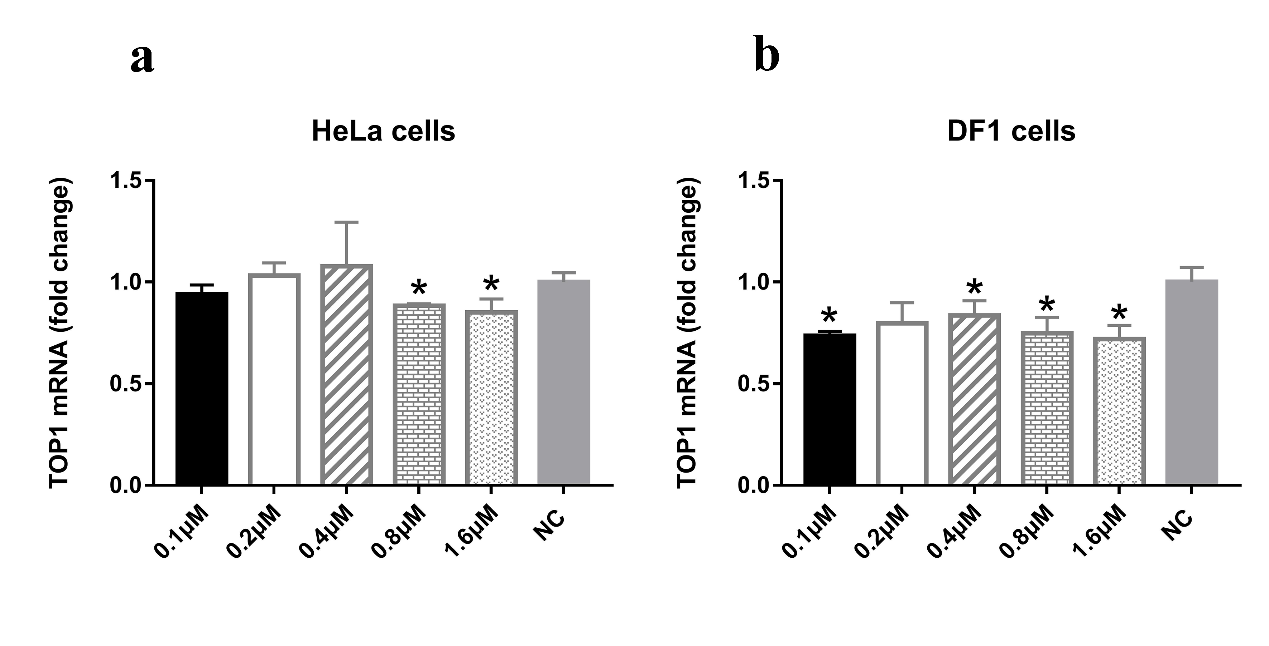


**Figure S1.** 2-5A downregulated the expression of TOPⅠ gene. Cells were transfected with 2-5A in different concentration. TOPⅠ was detected at 6 hours post transfection. (**a**) HeLa cells. (**b**) DF1 cells.


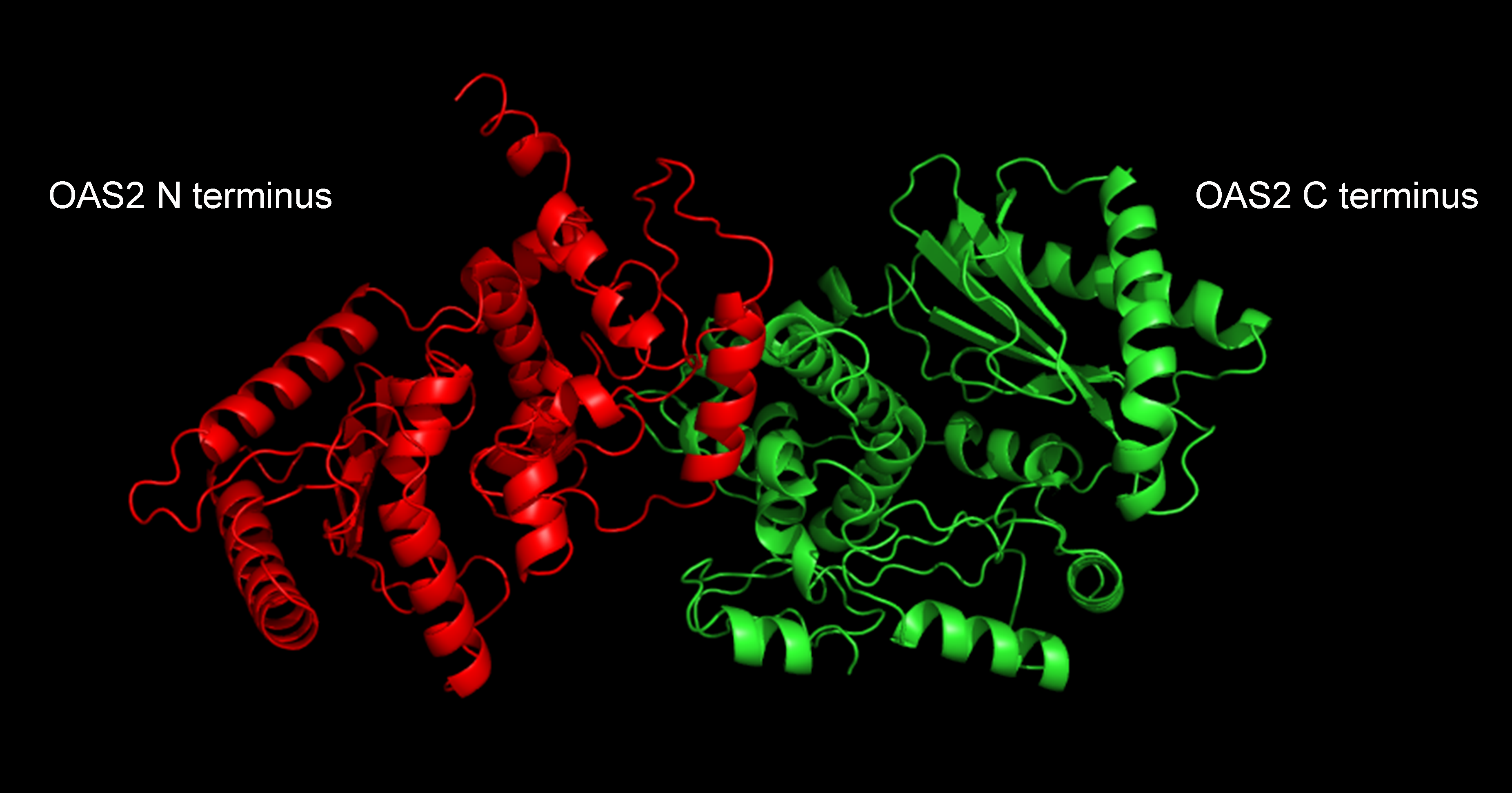


**Figure S2.** Predicted structure of human OAS2 protein. N terminus (left) and C terminus (right) of OAS2 were colored in red and green respectively.


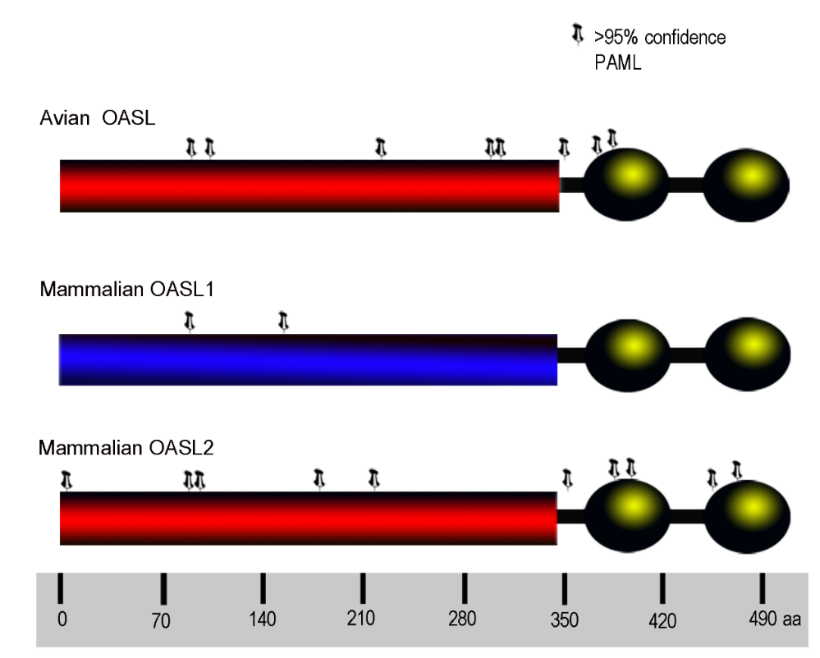


**Figure S3.** Distribution of positively selected sites for the OASL gene. Sequence alignment was carried out using the PRANK software (version 140603) and positively selected sites were predicted using PAML software (version 4.9). Sites displaying significant ω values were shown. Sequences for avian OASL, mammalian OASL1 and OASL2 refer to ostrich OASL, alpaca OASL1 and shrew OASL2, respectively.


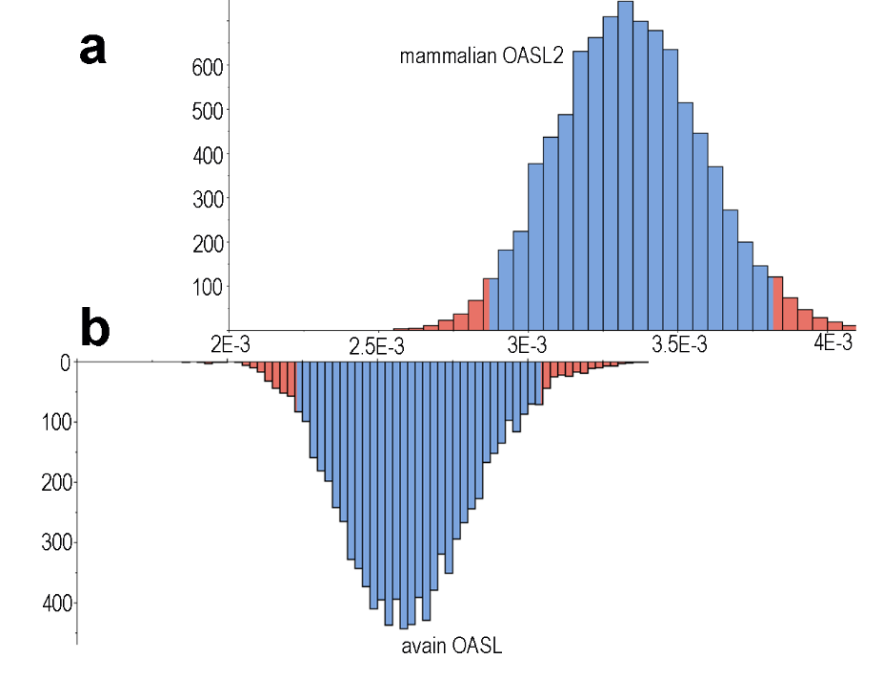


**Figure S4.** Evolutionary rate of mammalian OASL2 and avian OASL genes. Sequence alignment was performed by PRANK software (version 140603) and evolutionary rate was calculated using BEAST software (version 2.47). (**a)** Mammalian OASL2. (**b)** Avian OASL. It is clear that mammalian OASL2 has evolved faster than avian OASL.


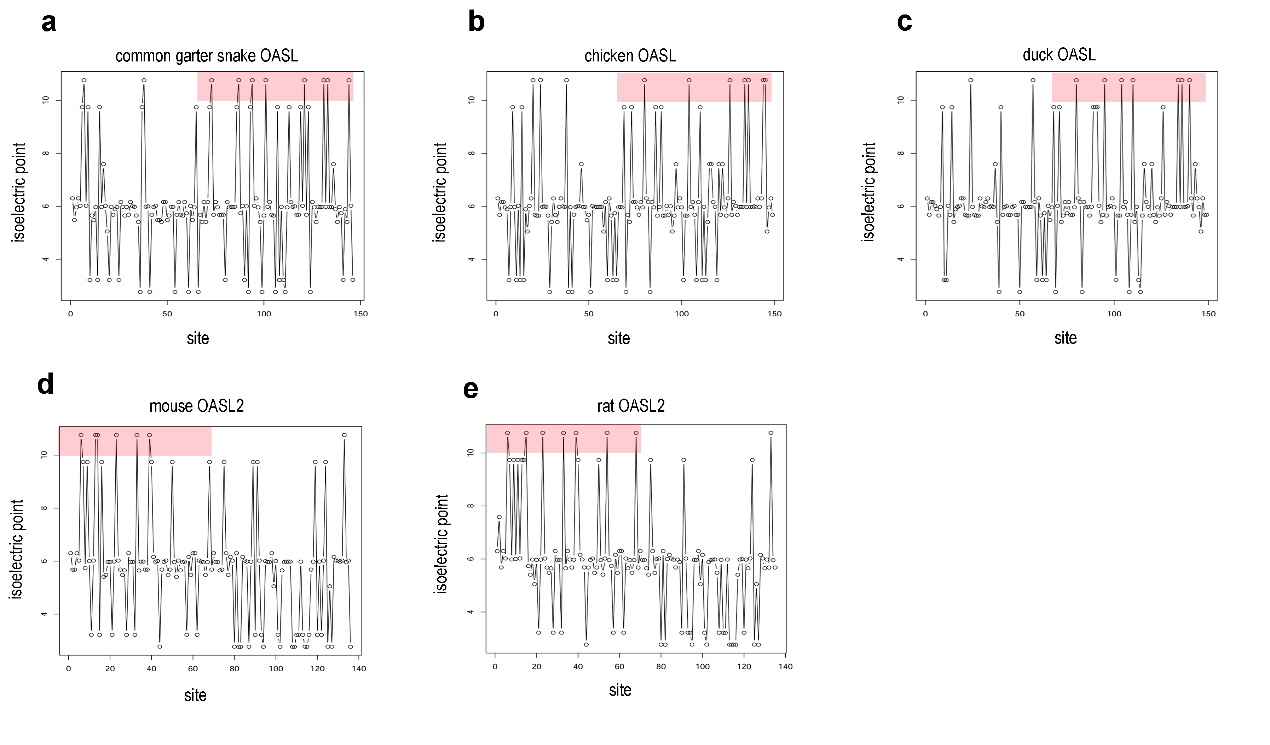


**Figure S5.** Isoelectric point of residues in two UBL domains of OASLs (C terminus of OASL protein; ~ 150 residues). (**a**-**c)** Reptilian and avian UBL domains. Common garter snake, duck and chicken OASLs were selected as reptilian and avian OASL representatives. **(d**-**e)** Mammalian UBL domains. Mouse and rat OASL2 were selected to represent mammalian OASL2. Avian and reptilian OASL proteins contain more basic residues (pink box) in the second UBL domain, while mammalian OASL proteins prefer to harbor basic residues (pink box) in the first UBL domain.
